# Developing an Internship Preparedness Program for Final Year Medical Students

**DOI:** 10.15694/mep.2018.0000219.1

**Published:** 2018-09-21

**Authors:** Bushra Nasir, Kate Jurd, Sheila Cook, Marcella Kwan, Remo Ostini

**Affiliations:** 1 The University of Queensland; 2 The University of Queensland

**Keywords:** Internship, Medical Students, Medical Education, Active-Learning, Work readiness

## Abstract

This article was migrated. The article was marked as recommended.

**Introduction**: Medical students undertake extensive training yet often feel they lack the practical non-medical skills required for successful transition to internship. While research provides evidence for student perceptions and experiences regarding internship, there is little information regarding how students can be ‘taught’ work-readiness through learning non-medical skills.

**Methods**: By reducing cognitive load when structured using a flipped classroom method, active learning resources may provide an effective approach to prepare medical students to be work-ready interns. Using a blended instructional method, the University of Queensland’s Rural Clinical School (UQRCS) integrated face-to-face and online learning, to develop an Intern Preparedness program.

**Discussion**: The Intern Preparedness program promotes student interaction in a range of active learning tasks to improve learning and engagement in a difficult and often neglected area of professional training. The program focused on providing knowledge and skills to increase competency in non-medical skills including time management on the ward, patient-focused prioritisation of tasks, and clinical conversations with the healthcare team.

**Conclusion**: This program has become an integral part of the student learning experiences at the UQRCS as it continues to elevate student preparedness for internship. The program has become a fundamentally important aspect of improving cognitive skills such as critical thinking and reasoning, as well as soft skills, which are all essential for successful transitions to internship. A very high uptake and completion of program activities provided further incentive for program developers to continue its improvements over time.

## Introduction

The transition from being a medical student to becoming an intern is notoriously demanding. Medical schools have an important role in supporting this important transition. Many medical schools and universities are reforming curriculum and providing enhanced training and learning ( Aretz, 2003; Elnicki *et al.*, 2017; Heidemann *et al.*, 2018; Stewart *et al.*, 2018). The medical curriculum ensures that medical students amass vast clinical knowledge with a solid grounding in medical science. However, this does not formally translate to providing them with the practical skills and basic ward-work knowledge required to become an effective junior doctor. Furthermore, despite these reforms and the associated enhanced training, medical students are still experiencing anxiety, and often feel that they are not prepared for internship, lacking confidence in their abilities and training ( Evans *et al.*, 2004; Gome *et al.*, 2008; Brennan *et al.*, 2010; Sturman *et al.*, 2017).

The importance of non-medical skills in facilitating successful transition to internship, work readiness and professional competence is well-recognised ( Epstein and Hundert, 2002; Caballero *et al.*, 2011; Scicluna *et al.*, 2014; Drummond *et al.*, 2016; Elnicki *et al.*, 2017). Furthermore, the transition to practice challenge is not unique to medicine, also occurring, for example, in pharmacy ( Fejzic and Barker, 2015), dentistry ( Dawson *et al.*, 2016), and among veterinarians ( Cake *et al.*, 2016).

Students have indicated that the stress they experience may be lightened by better leadership and support from their medical school during this significant learning occasion ( Scicluna *et al.*, 2014). While there is considerable evidence around what students think about being prepared for internship ( Hill *et al.*, 1998; Eley, 2010; Kelly *et al.*, 2011; Scicluna *et al.*, 2012; Scicluna *et al.*, 2014), it remains an open question as to whether a medical student can be ‘taught’ to be ready for their internship, particularly when it comes to non-medical skills.

The learning framework in medical schools has been criticised for encouraging recall instead of understanding and the capacity to solve new problems, with little emphasis on communication skills and teamwork ( Sefton, 2004). Such an approach works against the development of the non-medical skills that are a prominent component of internship.

With respect to learning theory, despite the range of different teaching methods available, medical educators still consider the memorisation of what is taught to be essential for medical students ( Qiao *et al.*, 2014). This learning task causes a large intrinsic cognitive load, due to the volume of material that must be learned and creates an immense learning challenge ( Sweller, 1994). Based on the assumption that people have limited working memory, cognitive load theory (CLT) has been designed to guide instructional design and promote learning by demonstrating the value of reducing the extraneous cognitive load that is due to the manner in which learning material is presented ( Sweller *et al.*, 1998). Reducing extraneous cognitive load leaves more working memory available for application to intrinsic learning tasks. Research has shown that CLT can provide a useful framework for the design and presentation of learning materials in medical education ( Qiao *et al.*, 2014).

Near-peer education has also been identified as valuable in advanced medical training ( de Menezes and Premnath, 2016), including the transition from preclinical to clinical training ( Knobloch *et al.*, 2018). Near-peer teaching uses teachers who have recently completed the educational tasks that students are facing and may be particularly suited to transition periods of increased stress ( Knobloch *et al.*, 2018).

In addition to teaching the curriculum, medical schools have an obligation to create and use effective teaching and learning resources to enhance and support the medical curriculum, as well as stimulate the student learning experience. An interactive learning program that is based on active learning, designed using principles of CLT, and incorporates collaborative peer-to-peer and near-peer teaching could be an effective method to prepare medical students for work as an intern.

Here we explore the development of a blended learning model that is designed to improve student interaction and engagement during learning to enhance their preparation for internship. This model emphasises the socialisation and communication qualities of face-to-face interactions and combines them with the technological possibilities of interactive and stimulating online activities. A program created in this manner will ensure purposeful interactions with content, teachers and peers.

## Flipped Classrooms and Blended Learning

There has been a recent trend to move away from the traditional classroom lecture to a more student focused approach based on active learning and engagement ( Abdel Meguid and Collins, 2017), adopting a constructivist pedagogical approach to ensure that the learner is always doing something that is central to active learning ( Beetham and Sharpe, 2007).

Digital technologies also have an impact on the instructional strategies that can be used to improve the student learning experience. In this context, blended learning programs have been introduced, where there is a combination of online and face-to-face activities. Focussing on one side of this blend the flipped classroom has become increasingly popular in an attempt to improve the traditional lecture. Advocates of the flipped classroom focus on improving the face-to-face session by using approaches that encourage higher order thinking and active participation in the classroom only.

The approach uses an educational model in which the standard lecture and homework elements of a course are ‘reversed’ or flipped. The homework component is now completed before the lecture and consists of a range of materials including, pre-recorded lectures, voice over PowerPoint, journal articles, YouTube videos, or textbook readings.

The main goal of the flipped classroom is to ensure the students complete the foundational learning - that is, having an understanding or good grasp of the topic - before they attend the lecture. This is to enable students to contribute at a higher level during the in-class activities and so develop their problem solving, analytical and synthesis skills under the guidance of the teacher. The teacher’s in-class delivery changes from didactic presentation or transmission of facts to a facilitator of learning through active, collaborative and interactive activities.

The flipped classroom has underpinnings in both the constructivist and social learning theory as it permits and encourages students to view learning as an active and social process ( Moraros *et al.*, 2015). In comparison, the typical traditional lecture evokes a passive consumption of knowledge where students are not challenged to construct knowledge or reflect on their learning. An important limiting factor of the traditional lecture is that it does not promote higher order thinking, as described in Bloom’s taxonomy of learning; specifically analysis, synthesis and evaluation ( Sherbino *et al.*, 2015).

**Figure 1.**
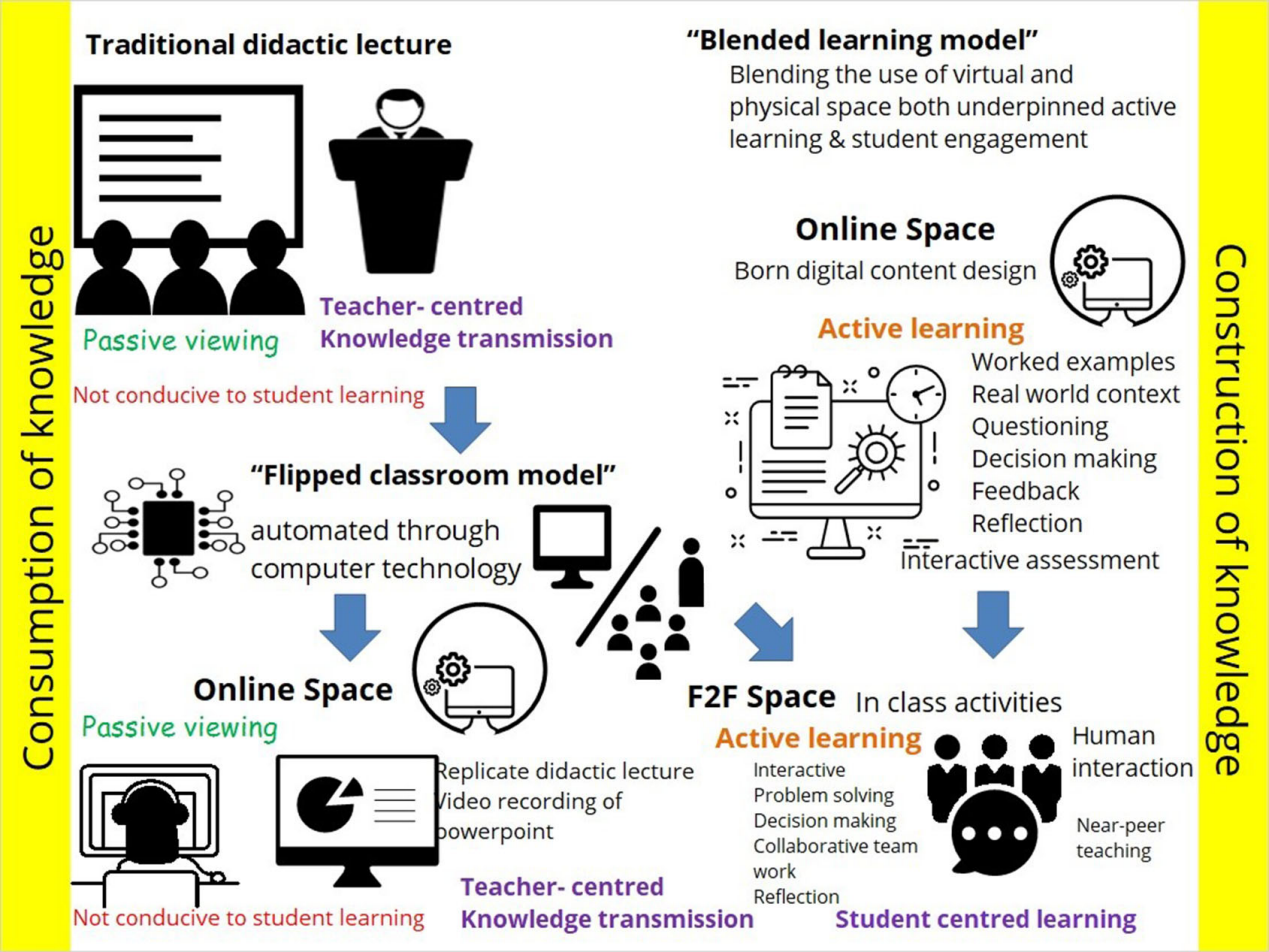
Moving to a blended model based on active learning ( Jurd, 2015)

This trend towards ‘flipping the classroom’ has seen a proliferation of video lectures available for students to access online. However, these examples of content delivery also evoke a passive consumption of content and superficial learning, which is often little different from the traditional classroom lecture (see Figure 1). Questions to consider in this approach are: 1. How well does the online content equip the students for the classroom discussion; 2. What type of content is being presented and what instructions are given to the student to manipulate the content; and 3. How are students interacting with the content to get the most out of the online learning experience?

If the student is not able to actively process the online materials or is not given instruction on what to do with the online content then it will have a limited effect on learning and transfer of knowledge to different contexts. If flipping then fails, it is not that this instructional strategy has failed but that the online component has not been designed effectively to promote interaction and engagement with the content. The true purpose of flipping the classroom is to facilitate students’ interaction with the content and this will not be accomplished simply by having content accessible online rather than having this content provided in a classroom setting.

## Implementing a Flipped Classroom in Developing an Intern Preparedness Program

At the conclusion of the medical degree, students will have acquired clinical knowledge and skills at a level that ensures a successful performance in the examination setting. Although students will typically have spent time in wards observing clinicians and participating in ward rounds, they are rarely trained in the non-medical skills that are required to be an effective junior doctor. Much is made of becoming “work-ready”, however, there is limited formal training for university students to develop the professional and workplace skills that ensure a successful transition from medical school to the busy and demanding work environment of a hospital.

## Motivation

In 2014, the clinical teaching team at a regional medical school site (University of Queensland Rural Clinical School: UQRCS) in Queensland, Australia began developing a program explicitly focusing on preparing students for their role as interns on hospital wards. The program’s development was a response to clinical staff observing that new medical graduates were stressed by the demands put on them by the busy ward environment and overwhelmed by the need to demonstrate skills that they were not trained in during medical school. Those skills include time management in the hospital, prioritisation of clinical tasks, managing regular distractions from pagers, phone calls and ward staff, as well as constant uncertainty and the need to make timely clinical decisions.

The demands causing new interns difficulty are inherent in the busy day-to-day work environment of a hospital ward and are immediately imposed on day one of work. The implicit assumption is that early graduates have these skills from day one. As more senior clinicians are focussed on making complex clinical decisions and managing their own workload, the more basic ward tasks are expected of the new graduates. In addition, limited supervision may be available for junior doctors in performing unfamiliar but routine tasks like paperwork, clinical conversations with other team members, leading team discussions and family meetings.

If junior doctors are not well-prepared for this demanding environment, the potential consequences are manifold. For patients, this can mean that important investigations can be missed or delayed and can contribute to an increased likelihood of prescribing errors ( Coombes *et al.*, 2008). Discharge summaries and important clinical conversations with allied health, general practitioners (GPs) and senior doctors may also be missed or delayed. For the nurses, the consequences are that their patients are not seen in a timely fashion. For the junior doctors themselves, this can lead to: Poorer relationships with the nurses and allied health clinicians on the ward; poorer impression being made on senior clinicians, which may influence the level of clinical and emotional support junior doctors receive from their supervisors; poor self-esteem, failing confidence and high risk for anxiety and depression; and perception of limited career options if the impression on senior clinicians is poor.

## Program development and evaluation

In an effort to address the perceived need for medical graduates to be better prepared for the non-medical demands of internship, educators at UQRCS implemented a blended learning program using active learning pedagogy supported by digital technology. The online content was created from scratch, that is, born digital, and did not replicate or reproduce a passive didactic lecture in the online space. Instead, interactive elements, including questions (with immediate feedback) and decision making-tasks were embedded in the content.

The aim of the program was to provide final year medical students with a clearer understanding of seven key factors for effectively completing a ward round. These are:

• The role and tasks of an intern and each member in the clinical team on the wards
• Time management, self-direction, resourcefulness and independence in the hospital environment
• Patient-focussed prioritization of tasks
• Communication skills, including clinical handover, how to refer patients, as well as telephone and paging etiquette
• How to recognise one’s limitations and how to ask for help and learn from others
• How to build relationships with other junior doctors in the hospital
• How to cope with uncertainty and adversity

The program was developed as a series of online learning modules that prepared the medical students for a practical, face-to-face ward round tutorial.

## Online content

Online learning modules were created as interactive and practical examples of clinical scenarios that might occur on a ward round. They included examples of “how to” and “how not to” undertake specific, non-medical tasks and allowed students to reflect on the consequences of doing things well and not so well. The examples in the learning modules were patient-focussed and provided an opportunity for students to understand the bigger picture of the hospital wards and the important role of the intern in the ward round.

The online learning module content was specifically designed to introduce new concepts through a series of worked examples and demonstrations that allowed students to process the information through examination and observation. The online instructional strategies were guided by cognitive theory and content elements were presented in a format designed to reduce cognitive load and build schema for long term memory ( Sweller, 2004). This included the inclusion of authentic learning tasks, guided instruction and supportive information to assist in knowledge acquisition and the transfer of learning to new contexts.

## Face-to-face content

The face-to-face tutorials were designed to facilitate discussion between the medical students and the junior doctors who were an integral part of tutorial delivery. Tutorials were built around a small group structure in order to promote discussion and included dedicated time for question and answer sessions. This allowed for the development of relationships between students and junior doctors whose role includes mentoring and professional support for new doctors in the wards. Recruiting junior doctors to assist with tutorials strengthened the focus on practical strategies from doctors who had recently experienced the scenarios for which the medical students were preparing. This approach helped to develop confidence in the medical students who could see these doctors as colleagues. The relationships that developed were therefore more horizontal than the relationships between the more senior doctors who provide formal teaching in the course of student clinical rotations.

## Online learning module content creation process

Content for the online learning modules was created by a subject matter expert - a senior medical officer and clinical educator. Each module was produced as a set of PowerPoint slides for adaptation by a learning technologist. The learning technologist converted this material for online use, based on active learning theory instructional design as described above. In addition to presenting the material in a way that did not overburden working memory, allowed active processing of information (schema) and transfer to long term memory ( Sweller, 2004), it was also important to consider the ‘modality effect’ ( Mayer and Moreno, 2003), using audio and visual channels to ensure maximum processing capacity.

In converting the subject matter expert material for online delivery, the learning technologist considered a number of factors. These included intuitive navigation through the material to facilitate ease of use. Visual design took content aesthetics into account, including such factors as the placement of elements on the screen, font and colour selection. It was important for the appearance of the content to be uncluttered in order to reduce extraneous load ( Mayer and Moreno, 2003) and ensure that learners were not overwhelmed by the experience.

User learning design principles also supported taking the included content activities from real world contexts and presenting them as worked examples with guided instruction ( Sweller, 2006). That is, authentic ward scenarios illustrating the non-medical ward skills of an expert clinician provided students with worked solutions to unfamiliar but common situations. This provided applicability and relevance and more meaning, which could be expected to assist in the transfer of knowledge to other contexts and importantly, to work practice.

The structure of the module content provided a sequential flow of information dispersed with activities designed to retain student attention and allow students to move from passive observation to active engagement. Emphasis was placed on chunking content into learning segments that allowed students to reflect and synthesize information during each phase of the learning activity.

A number of factors guided the creation of the content’s interactiveness. The first consideration was the instruction given to the students. This was framed in terms of the learning outcomes of the online activities and considered what the student needed to do with the content. Subsequently, activities were designed as if they were being designing for an interactive classroom. The use of questioning and processing points throughout the content helped create activities where the student needed to ‘think’, that is, analyse, synthesise and solve problems ( Adams, 2015). As a result, the online learning activities required a learner to think about what was being presented and make a decision, answer a question pertaining to the material, and justify ‘why’ it was so. At this point it was important to provide feedback and to explain the reasoning behind both the correct and incorrect answers to ensure students understood their mistakes and their correct answers and did not simply identify the right answer by guessing. In this way, the embedded activities provided students with opportunities to enhance their non-medical decision-making skills.

**Figure 2.**
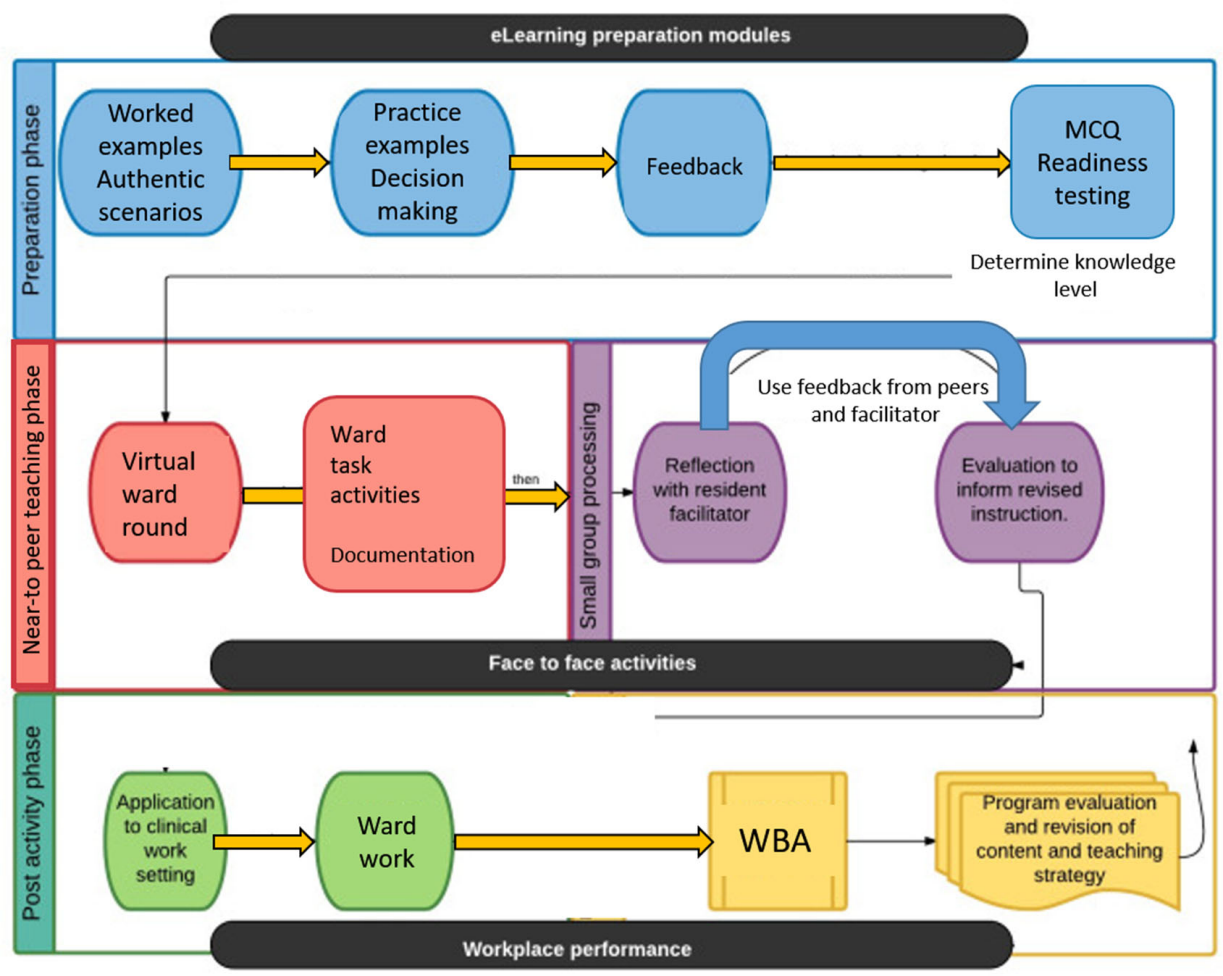
A flipped model based on active learning pedagogy ( Jurd, 2014)

The ward management skills online modules that were created through the process described above were directly linked to the activities of the face-to-face tutorials. The foundation knowledge and skills gained by completing the online modules enabled learners to effectively participate in the mock ward round presented in the tutorials and to confidently manage the tasks. The expectation is that students will subsequently be able to retrieve this knowledge to solve the ‘real world’ problems presented in the face-to-face activities, learning by doing. This is a more sound pedagogical approach, which provides students with an avenue to practice the new knowledge and skills in the mock ward round workshop scenario and then reflect on their performance during junior doctor small group discussions. The ‘in class’, face-to-face workshop activities created for this program ensure that students learn through participation in a shared community of practice ( Lave and Wenger, 1991). and in their rehearsal or practice of ‘real-world’ non-medical decision making they learn how to think and act in similar situations but with differing presentations or context. The junior doctor small group discussions added an extra learning dimension to the activity, providing students with guided self-reflection, constructive feedback and suggested ways to enhance their skills and performance once they transition to the workplace. Figure 2 provides a framework that shows the major components of the intern preparedness program and how they are expected to relate to workplace performance.

The focus of the education in both the online and face-to-face content is on cognitive skills such as critical thinking and reasoning as well as the soft skills that are fundamentally important for intern preparation (work readiness) and success in future transitions in training.

Students were given access to the online learning modules a week prior to the face-to-face tutorial. The modules were made available on the university’s learning management system and UQRCS educators were able to monitor completion through that system’s grade centre. This allowed them to send students reminders to complete the modules as the workshop approached. Literature suggests that uptake and completion of online modules is relatively low ( Smith, 2005), indicating that students have low self-regulatory learning skills. However, perhaps due to the nature of the topic (intern preparedness), final year students were intrinsically motivated to complete both online and the in-class activities, with 83-100% completion rates for the pre-class online learning modules.

## Program Improvements

Delivering this program over a number of years has enabled a process of program development and improvement driven by participant evaluation of all program components. Students evaluated each online module and the face-to-face tutorials separately, as they completed each activity. Junior doctor participants in the face-to-face tutorials also provided feedback and evaluation of this activity. Program evaluation and feedback was purposefully collected using Likert scale survey and open-ended questions specifically designed for each program component. The regular collection of evaluation data and feedback produced changes to both online module and face-to-face tutorial content and methods of delivery. Changes included adding audio content to online modules, providing more structured time to complete tasks in the face-to-face tutorial, and using different ward scenarios in the face-to-face tutorial than for the online modules.

## Conclusion

Preparing medical students to begin practicing as doctors is a notoriously demanding task for both students and educators ( Brennan *et al.*, 2010; Sturman *et al.*, 2017). In that context, student’s learning of the non-medical skills required to succeed in a typical hospital ward-based internship is often overlooked in the formal education process. This is particularly the case when students are clinically trained in the typical ward-based rotation process, where it is assumed that students will learn the necessary skills, as if by osmosis, simply by being in that environment. Evidence suggests that this does not happen ( Walker *et al.*, 2013; Sturman *et al.*, 2017). This leaves a pedagogical challenge, to design an approach to conveying these skills as part of a formal education program in a way that minimises the additional learning demands on students and maximises the prospect of the skills being learned in a readily transferable manner.

This paper describes the contribution of cognitive load theory to this task and the way it is applied through principles of instructional design to produce an educational program to assist soon to graduate medical students prepare for the rigours of internship. The resulting program took a blended learning approach to the flipped classroom model to produce a number of interactive online learning resources built around real life ward scenarios. The interactive component of the resources engaged students in thinking about the scenarios and provided feedback on whether and why their responses were more or less appropriate for the questions being raised. The online scenarios prepared students for subsequent face-to-face tutorials that drew on the participation of junior doctors to demonstrate realistic ward scenarios, giving students an opportunity to put the skills they had learned in the online modules into practice in a safe environment. The face-to-face tutorials were designed to facilitate the transfer of the skills learned to subsequent internship. In this way, it is expected that students will be better prepared to manage the rigours of internship through learning necessary non-medical skills in an efficient, engaging and effective manner. Upon evaluation, this program can provide a model for other medical schools aiming to improve the transition to hospital-based practice for their students.

## Take Home Messages

• Students’ learning of non-medical skills is essential for preparation for internship, alongside formal medical training.
• Reducing cognitive load by developing active learning resources using a blended learning approach can enhance intern preparedness through the learning of non-medical skills.
• Designing a program that provides a blended learning approach to the flipped classroom model for students to interactively engage and learn essential skills as part of their typical ward-based rotation training has provided an effective and efficient model for preparing interns to be work-ready.

## Notes On Contributors

Bushra Nasir is a Research Fellow at the University of Queensland Rural Clinical School, Toowoomba, Queensland, Australia.

Kate Jurd is an eLearning Specialist Fellow at the University of Queensland Rural Clinical School, Toowoomba, Queensland, Australia.

Dr Sheila Cook is the Staff Endocrinologist at Toowoomba Hospital, Toowoomba, Queensland, Australia and Senior Lecturer at the University of Queensland Rural Clinical School, Toowoomba, Queensland, Australia.

Marcella Kwan is an Epidemiologist at the University of Queensland Rural Clinical School, Toowoomba, Queensland, Australia.

Remo Ostini is a Senior Research Fellow at the University of Queensland Rural Clinical School, Toowoomba, Queensland, Australia.

## References

[ref1] Abdel Meguid E. and Collins M. (2017) Students’ perceptions of lecturing approaches: Traditional versus interactive teaching. Advances in Medical Education and Practice.pp.229–241. 10.2147/AMEP.S131851 28360541 PMC5364003

[ref2] Adams N. (2015) Bloom’s taxonomy of cognitive learning objectives. Journal of the Medical Library Association. 103(3), pp.152–3. 10.3163/1536-5050.103.3.010 26213509 PMC4511057

[ref3] Aretz H. (2003) How good is the newly graduated doctor and can we measure it? Med J Aus. 178(4), pp.147–148. 10.5694/j.1326-5377.2003.tb05126.x 12580736

[ref4] Beetham H. and Sharpe F. (eds.) (2007) Rethinking Pedagogy for a digital age. London, New York: Taylor Francis Group. 10.4324/9780203961681

[ref5] Brennan N. Corrigan O. Allard J. Archer J. (2010) The transition from medical student to junior doctor: today’s experiences of Tomorrow’s Doctors. Med Educ. 44(5), pp.449–58. 10.1111/j.1365-2923.2009.03604.x 20518984

[ref6] Caballero C. L. Walker A. and Fuller-Tyszkiewicz M. (2011) The Work Readiness Scale (WRS): Developing a measure to assess work readiness in college graduates. Journal of Teaching and Learning for Graduate Employability. 2(2), pp.41–54. 10.21153/jtlge2011vol2no1art552

[ref7] Cake M. A. Bell M. A. Williams J. C. Brown F. J. L. (2016) Which professional (non-technical) competencies are most important to the success of graduate veterinarians? A Best Evidence Medical Education (BEME) systematic review: BEME Guide No. 38. Medical Teacher. 38(6), pp.550–563. 10.3109/0142159X.2016.1173662 27145182

[ref8] Coombes I. D. Stowasser D. A. Coombes J. A. and Mitchell C. (2008) Why do interns make prescribing errors? A qualitative study. The Medical Journal of Australia. 188(2), pp.89–94. 18205581 10.5694/j.1326-5377.2008.tb01529.x

[ref9] Dawson L. J. Mason B. G. Bissell V. and Youngson C. (2016) Calling for a re-evaluation of the data required to credibly demonstrate a dental student is safe and ready to practice. European Journal of Dental Education. 21(2), pp.130–135. 10.1111/eje.12191 27027651 PMC5396269

[ref10] de Menezes S. and Premnath D. (2016) Near-peer education: a novel teaching program. International Journal of Medical Education. 7, pp.160–167. 10.5116/ijme.5738.3c28 27239951 PMC4885635

[ref11] Drummond I. Sheikh G. Skinner J. and Wood M. (2016) Exploring the feasibility and acceptability of using tactical decision games to develop final year medical students’ non-technical skills. Medical Teacher. 38(5), pp.510–514. 10.3109/0142159X.2016.1150979 27008190

[ref12] Eley D. (2010) Postgraduates’ perceptions of preparedness for work as a doctor and making future career decisions: support for rural, non-traditional medical schools. Education for Health. 23(2), p.374. 20853241

[ref13] Elnicki D. M. Aiyer M. K. Cannarozzi M. L. Carbo A. (2017) An Entrustable Professional Activity (EPA)-Based Framework to Prepare Fourth-Year Medical Students for Internal Medicine Careers. J Gen Intern Med. 32(11), pp.1255–1260. 10.1007/s11606-017-4089-8 28634908 PMC5653547

[ref14] Epstein R. M. and Hundert E. M. (2002) Defining and assessing professional competence. The Journal of the American Medical Association. 287(2), pp.226–235. 10.1001/jama.287.2.226 11779266

[ref15] Evans D. Wood D. and Roberts C. (2004) The effect of an extended hospital induction on perceived confidence and assessed clinical skills of newly qualified pre-registration house officers. Med Educ. 38(9), pp.998–1001. 10.1111/j.1365-2929.2004.01908.x 15327682

[ref16] Fejzic J. and Barker M. (2015) ‘The readiness is all’ - Australian pharmacists and pharmacy students concur with Shakespeare on work readiness. Pharmacy Education. 15(1), pp.76–82.

[ref17] Gome J. Paltridge D. and Inder W. (2008) Review of intern preparedness and education experiences in General Medicine. Intern Med J. 38(4), pp.249–53. 10.1111/j.1445-5994.2007.01502.x 18298561

[ref18] Heidemann L. A. Walford E. Mack J. Kolbe M. (2018) Is There a Role for Internal Medicine Residency Preparation Courses in the Fourth Year Curriculum? A Single-Center Experience. J Gen Intern Med. 10.1007/s11606-018-4620-6 PMC6258616 30094763

[ref19] Hill J. Rolfe I. Pearson S. and Heathcote A. (1998) Do junior doctors feel they are prepared for hospital practice? A study of graduates from traditional and non-traditional medical schools. Med Educ. 32, pp.19–24. 10.1046/j.1365-2923.1998.00152.x 9624395

[ref20] Jurd K. (2014) A flipped model based on active learning pedagogy. How engaging is your eLearning content? Available at: https://katejurd.wordpress.com/2014/11/23/designing-for-online-engagement/

[ref21] Jurd K. (2015) Blended learning framework to teach clinical reasoning. Learning Design: Technology enhanced learning in Medical Education[Blog]. Available at: https://katejurd.wordpress.com/2015/12/28/flipped-learning-framework-to-teach-clinical-reasoning/

[ref22] Kelly C. Noonan C. and Monagle J. (2011) Preparedness for internship: a survey of new interns in a large Victorian Health Service. Australian Health Review. 35, pp.146–151. 21612725 10.1071/AH10885

[ref23] Knobloch A. C. Ledford C. J. W. Wilkes S. and Saperstein A. K. (2018) The Impact of Near-Peer Teaching on Medical Students’ Transition to Clerkships. Family Medicine. 50(1), pp.58–62. 10.22454/FamMed.2018.745428 29346691

[ref24] Lave J. and Wenger E. (1991) Situated learning: Legitimate peripheral participation. Cambridge: Cambridge University Press. 10.1017/CBO9780511815355

[ref25] Mayer R. E. and Moreno R. (2003) Nine ways to reduce cognitive load in multimedia learning. Educational Psychologist. 38(1), pp.43–52. 10.1207/S15326985EP3801_6

[ref26] Moraros J. Islam A. Yu S. Banow R. and Schindelka B. (2015) Flipping for success: evaluating the effectiveness of a novel teaching approach in a graduate level setting. BMC Med Educ. 15, p.27. 10.1186/s12909-015-0317-2 25884508 PMC4363198

[ref27] Qiao Y. Shen J. Liang X. Ding S. (2014) Using cognitive theory to facilitate medical education. BMC Medical Education. 14, p.79. 10.1186/1472-6920-14-79 24731433 PMC3989791

[ref28] Scicluna H. A. Grimm M. C. Jones P. D. Pilotto L. S. (2014) Improving the transition from medical school to internship - evaluation of a preparation for internship course. BMC Medical Education. 14, p.23. 10.1186/1472-6920-14-23 24485072 PMC3913947

[ref29] Scicluna H. A. Grimm M. C. O’Sullivan A. Harris P. (2012) Clinical capabilities of graduates of an outcomes-based integrated medical program. BMC Med Educ. 12, p.23. 10.1186/1472-6920-12-23 22540877 PMC3372445

[ref30] Sefton A. J. (2004) New approaches to medical education: an international perspective. Medical Principles and Practice. 13(5), pp.239–248. 10.1159/000079521 15316255

[ref31] Sherbino J. Chan T. and Schiff K. (2015) The reverse classroom: lectures on your own and homework with faculty. Cjem. 15(3), pp.179–181. 10.2310/8000.2013.130996 23663466

[ref32] Smith P. (2005) Learning preferences and readiness for online learning. Journal of Educational Psychology. 25(1), pp.3–12. 10.1080/0144341042000294868

[ref33] Stewart M. K. Henry R. C. Ehrenfeld J. M. and Terhune K. P. (2018) Utility of a Standardized Fourth-Year Medical Student Surgical Preparatory Curriculum: Program Director Perceptions. J Surg Educ. 75(3), pp.639–643. 10.1016/j.jsurg.2017.09.004 29306578

[ref34] Sturman N. Tan Z. and Turner J. (2017) “A steep learning curve”: junior doctor perspectives on the transition from medical student to the health-care workplace. BMC medical education. 17, p.92. 10.1186/s12909-017-0931-2 28549459 PMC5446747

[ref35] Sweller J. (1994) Cognitive load theory, learning difficulty, and instructional design. Learning and Instruction. 4, pp.295–312. 10.1016/0959-4752(94)90003-5

[ref36] Sweller J. (2004) Instructional design consequences of an analogy between evolution by natural selection and human cognitive architecture. Instruct Sci. 32(1/2), pp.9–31. 10.1023/B:TRUC.0000021808.72598.4d

[ref37] Sweller J. (2006) The worked example effect and human cognition. Learning and Instruction. 16, pp.165–69. 10.1016/j.learninstruc.2006.02.005

[ref38] Sweller J. Merrienboer J. and Paas F. (1998) Cognitive Architecture and Instructional Design. Educational Psychology Review. 10(3), pp.251–96. 10.1023/A:1022193728205

[ref39] Walker A. Yong M. Pang L. Fullarton C. (2013) Work readiness of graduate health professionals. Nurse Educ Today. 33(2), pp.116–22. 10.1016/j.nedt.2012.01.007 22336479

