# Supplementary material for: Developing an Internship Preparedness Program for Final Year Medical Students
Source: MedEdPublish (2016). 2018 Sep 21;7:219. [Version 1] doi: 10.15694/mep.2018.0000219.1 (PMC10704419; doi:10.15694/mep.2018.0000219.1)
Supplement: Peer review history

# Reviewer report: Reviewer response for version 1

**DOI:** 10.21956/mep.19674.r29382

**Published:** 2019-03-15

**Authors:** Richard Hays

**Affiliations:** 1 James Cook University

This review has been migrated. The reviewer awarded 3 stars out of 5 This is an interesting paper that reports a blended learning approach to supporting senior medical students transition to internship, the first postgraduate year. This can be a difficult and stressful period, so such programs are needed. The paper could be strengthened by providing more information on why the topics were chosen, and explaining more about the delivery. Many of the concerns are very pra tidal (e.g. what to do whe the beeper goes off after hours) and some of this is better delivered in discussion-based sessions that include simulation. I look forward to reading the evaluation report, ideally from multiple perspectives (current students, recent graduates, employers etc). Those responsible for the final year of all medical programs may find this a worthwhile read.

Reviewer Expertise:

NA

No decision status is available

# Reviewer report: Reviewer response for version 1

**DOI:** 10.21956/mep.19674.r29381

**Published:** 2018-09-30

**Authors:** P Ravi Shankar

**Affiliations:** 1 American International Medical University

This review has been migrated. The reviewer awarded 4 stars out of 5 I enjoyed reading this well-presented article. Preparing interns for supervised practice is vital. More often than not, especially in the developing world, interns are left to cope on their own and the expectation is that they will learn on the job. Interns do get support from other interns, house officers and residents but this is not often incomplete. Many schools offer some orientation programs to interns just before the start of the internship. Over twenty-five years when I did my internship, I wish a training program described by the authors was in place. It would have made my transition much easier and less stressful. The online delivery of material and the face-to-face sessions with junior doctors have been well thought of and are based on sound learning principles. The emphasis on reducing cognitive load and facilitating comprehension is commendable. All medical educators will find this article of interest. With the reducing cost and wider availability of technology, educators in developing nations could develop similar programs.

Reviewer Expertise:

NA

No decision status is available

# Reviewer report: Reviewer response for version 1

**DOI:** 10.21956/mep.19674.r29383

**Published:** 2018-09-21

**Authors:** Virginia Randall

**Affiliations:** 1 Uniformed Services University of the Health Sciences

This review has been migrated. The reviewer awarded 4 stars out of 5 I applaud the authors for tackling a vexing problem, that of preparing medical students for the real world of medicine, i.e., inoculating them against the “hidden curriculum” and what the authors call learning by “osmosis.” The online learning using a flipped classroom model makes sense, as does the near peer interaction. The contributions of the e-educator are a nice example of what can be done to move a PowerPoint presentation to an engaging activity. I appreciate that they have had ongoing program evaluation both qualitative and quantitative, but would like to see some of that reflected by residents who are nearing the end of their training period.

Reviewer Expertise:

NA

No decision status is available
